# Identification of circulating Tfh/Th subsets as a biomarker of developed hospital-acquired pneumonia

**DOI:** 10.3389/fimmu.2025.1513939

**Published:** 2025-01-22

**Authors:** Yuan Peng, Tao Tao, Ni-Wen Yu, Chenyang Xu, Cheng Chen

**Affiliations:** ^1^ Intensive Care Unit, The First People’ ‘s Hospital of Kunshan Affiliated with Jiangsu University, Kunshan, China; ^2^ Respiratory Department, The First People’ ‘s Hospital of Kunshan Affiliated with Jiangsu University, Kunshan, China; ^3^ Respiratory Department, The First Affiliated Hospital of Soochow University, Suzhou, China

**Keywords:** hospital-acquired pneumonia, Tfh cell, Th, prognosis, PCT

## Abstract

**Background:**

This study aimed to explore the possible value of follicular helper T (Tfh) cells in hospital-acquired pneumonia (HAP).

**Methods:**

Flow cytometry was used to measure circulating Tfh and helper T cell (Th) cells in 62 HAP patients and 16 healthy individuals. HAP patients were further categorized into uncontrolled and controlled groups, in accordance with relevant guidelines. Subgroup analyses were additionally conducted based on the pathogen and the presence of bloodstream infections (BSIs) and the incidence of septic shock. Kaplan-Meier survival analysis and ROC analysis were performed to estimate the prognostic value of the combination of Tfh/Th ratios and PCT levels.

**Results:**

The Tfh/Th ratio was notably higher in uncontrolled HAP patients than in controls (P<0.05). Specifically, either the Klebsiella pneumoniae (K.p) -positive HAP or BSIs subgroups or septic shock subgroups showed significantly increased Tfh/Th ratios (P<0.05). PCT level in BSIs and septic shock subgroups was significantly increased. However, there were no significant differences in PCT level between K.p-infected and non-K.p-infected patients. So, the Tfh/Th ratio is a good supplement to PCT for distinguishing between the K.p and non-K.p groups. The Tfh/Th ratio also demonstrated a strong correlation with procalcitonin (PCT) levels (P<0.05). Accordingly, the combination of Tfh/Th and PCT could serve as a more effective predictive marker for HAP deterioration and survival prediction. HAP patients with a high Tfh/Th ratio along with high PCT levels had a lower 28-day survival rate.

**Conclusion:**

The circulating Tfh/Th ratio, instrumental in gauging the severity of patients with HAP, could be employed as a prognostic biomarker for HAP.

## 1 Introduction

In the intensive care unit (ICU), hospital-acquired pneumonia (HAP) remains the most common infection (1), which is usually associated with dysregulated immune responses. The immunopathogenesis of HAP is very complex that remains somewhat unclear. Resistance, or actions of the host to eradicate living microbes, in the lungs involves a combination of innate and adaptive immune responses triggered by air-space infection (2).

Follicular helper T (Tfh) cells are antigen-experienced T cells that can be found mainly in the B cell zone of secondary lymphoid organs and, to a lesser extent, in circulation (3). Tfh cells are known to be closely related to viral infections like HIV, but their relation to bacterial infections has not been widely studied. Usually, Tfh cells are identified by their constitutive expression of CXCR5 and play an indispensable role in forming and maintaining germinal centers for ongoing immune responses (4). Whether the Tfh cells could serve a role in bacterial pneumonia assessment and prognosis remains to be investigated.

In the present study, we investigated the frequency of Tfh cells and evaluated the ratio of Tfh/Th among subjects of HAP, which has been identified that the ratio of Tfh/Th could serve as a valuable biomarker for the management of HAP.

## 2 Materials and methods

### 2.1 Patients

A total number of 62 patients with HAP and 16 healthy individuals were recruited for our study. HAP was diagnosed according to the American Thoracic Society/European Respiratory Society (ATS/ERS) standards. HAP patients with one or more of the following conditions were excluded from this study: 1) asthma; 2) autoimmune diseases; 3) chronic obstructive pulmonary disease (COPD) and malignant disease. All protocols involving human participants were reviewed by the Ethics Committee of the First People’ ‘s Hospital of Kunshan Affiliated with Jiangsu University (Approval No.2021-06-022).

### 2.2 Definition

Patients fulfilling both of the subsequent conditions, in line with the ATS guidelines of HAP, were characterized as having uncontrolled pneumonia: 1) having two or more complications (e.g. septic shock, heart failure, acute respiratory distress and secondary infections) and 2) one or more of these complications did not improve after three days of active treatment (5, 6).

Bloodstream infection (BSIs) was defined by positive blood culture or cultures with an isolate of the same species grown in at least one blood culture bottle in a patient with systemic signs of infection (i.e. a patient who had evidence of one or more of the symptoms or signs, including fever (body temperature > 38 °C), hypothermia (body temperature < 36°C), chills, hypotension, oliguria, or high lactate levels) (7).

Septic Shock(SS) can be clinically identified by a vasopressor requirement to maintain a mean arterial pressure of 65 mm Hg or greater and serum lactate level greater than 2 mmol/L (>18 mg/dL) in the absence of hypovolemia by sepsis 3.0 (8).

### 2.3 Flow cytometry

All individuals were analyzed by flow cytometry to circulating Tfh cells (CXCR5+Foxp3-CD4+) and Th cells (CXCR5-FoxP3-CD4+). Flow cytometry analysis of the cell subsets in the peripheral blood was performed as previously described with modifications (6). 5 mL of venous blood was drawn from each patient into ethylenediaminetetraacetic acid (EDTA)-coated tubes (VACUETTE, Greiner Bio-One). Antibodies and flow cytometry cells were stained with fluorochrome-labelled APC-anti-CD45 (Catalog # HI30), FITC-anti-CD4 (Catalog # 550628) and PerCP-anti-CXCR5 (Catalog # RF8B2) antibodies, all from BD Biosciences (Heidelberg, Germany). Treg was detected with PE-anti-Foxp3 (Catalog # 259D/C7) and staining kit from BD Biosciences. Briefly, 100 µl of whole blood was incubated with appropriate concentrations of APC-anti-CD45, FITC-anti-CD4, PerCP-anti-CXCR5 for 30 min in the dark at room temperature. Staining for Foxp3 with PE-anti-Foxp3 was performed after fixation and permeabilization of the cells. Our operations were carried out by the same technician and executed in accordance with the manufacturer’s recommendations. Data were acquired using a BD FACS Calibur cytometer, and data analysis was performed using FlowJo 10.2 Software. The gating strategy was implemented based on the use of unstained controls, isotype controls, single-stain controls and the compensation, with each cell population clearly distinguished.

### 2.4 Laboratory testing

Lower respiratory tract secretion samples and blood samples were collected from each patient for microbiological examination. Serum was collected as well for procalcitonin (PCT) measurement. PCT was measured using Xin Chanye PCT automated immunoassays. The analytical sensitivity of all assays was <0.25g/L, as previously described (6).

### 2.5 Statistical analysis

All data in this study were expressed as mean± standard deviation (SD), and all statistical analyses were performed with SPSS version 19.0 (SPSS Inc.). Normal distribution data was analyzed by Unpaired Students t-test, while non-normal distribution data was committed to nonparametric tests. Comparison of multiple groups was performed with ANOVA if the data were normally distributed or Kruskal-Wallis test when the data did not distribute normally. Spearman correlation analysis was used to assess the correlation between PCT and Tfh/Th. Receiver operating characteristic (ROC) curves were calculated to select the cut-off level of ratio of Tfh/Th and PCT value indicating exacerbation of HAP. Kaplan-Meier survival analysis was performed to estimate the survival. All tests were two-sided, and a *P* value less than 0.05 was considered statistically significant.

## 3 Results

### 3.1 Patient characteristics

62 patients were recruited in our current study, with the baseline clinical characteristics listed in **Table 1**. Of the 62 recruited patients, 38 were male, and 24 were female, ranging from 18 to 85 years old. In terms of pathogen distribution, patients were mainly infected with Klebsiella pneumoniae (K.p), Acinetobacter baumannii, Escherichia coli, and Pseudomonas aeruginosa. In this cohort, 34 patients had uncontrolled HAP, 28 had controlled HAP, 21 were K.p-positive infected HAP, 11 were A.b-positive infected HAP and ten were complicated with BSIs, and 22 were complicated with septic shock. In addition, we enrolled 16 healthy individuals from the volunteer team as a healthy control group.

**Table 1:** Subject characteristics.

Characteristics	n=62
Age (y)	18-85
≧65	25
<65	37
Gender	
male	38
female	24
Underlying diseases	
Intracerebral hemorrhage	19
Cerebral embolism	10
Pelvic fracture	6
Renal failure	6
Pulmonary contusion	10
Brain contusion	11
Clinic status	
Uncontrolled	34
Controlled	28
Pathogens	
K.p	21
Non-K.p	41
A.b	11
Non-A.b	51
HAP with BSI	
Yes	10
No	52
HAP with Septic Shock	
Yes	22
No	40

K.p, Klebsiella pneumoniae; A.b, *Acinetobacter baumannii*; HAP, Hospital-acquired pneumonia; BSI, Bloodstream Infection.

### 3.2 The ratio of Tfh/Th is increased in uncontrolled HAP patients

**Figure 1:**
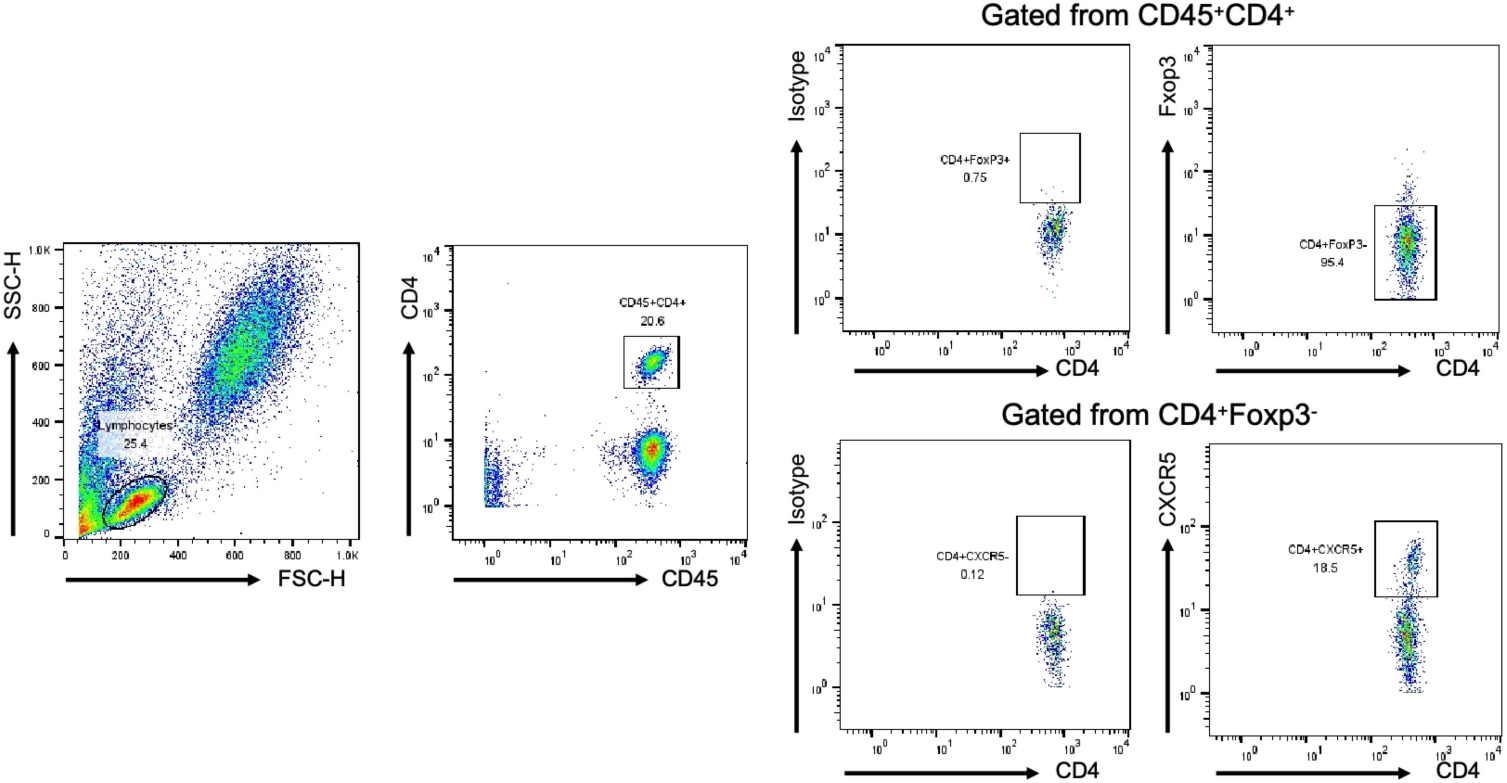
Analysis of Tfh and Th cells in all individuals by FCM. The lymphocyte population was gated according to cell size and complexity, CD45+CD4+T cell was gated from lymphocyte, Foxp3-CD4+T cell was gated from CD45+CD4+T cell, CXCR5+/-CD4+ T cell was gated from Foxp3-CD4+T cell. Representative dot plots are shown.

**Figure 2:**
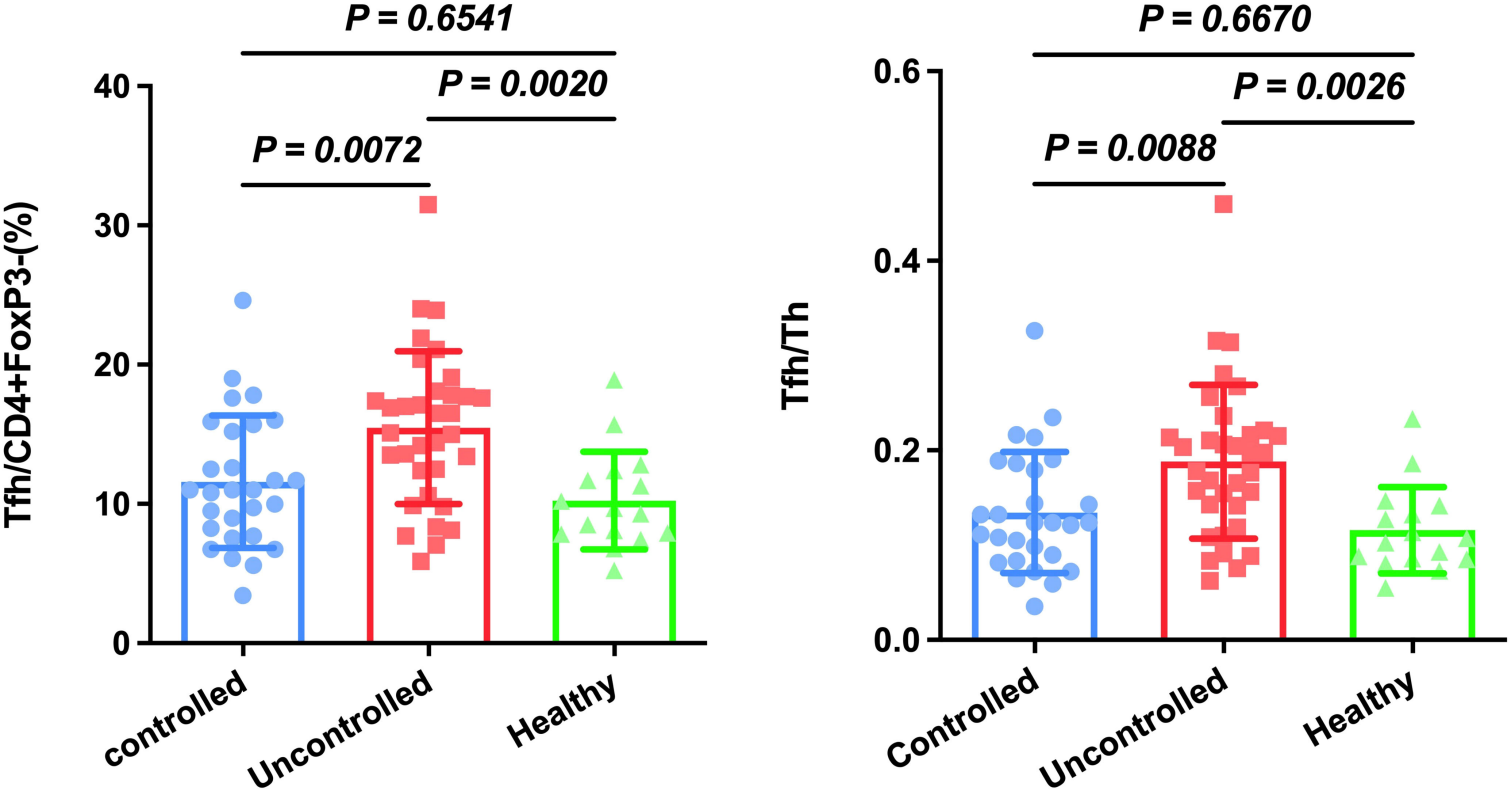
Circulating Tfh and Tfh/Th are increased in patients with uncontrolled HAP compared to the controlled HAP and healthy group. Results are presented as the mean with SD levels were compared by Anova analysis (*p* < 0.05).

TFH is reported to be closely associated with HAP in ICU patients (6). Firstly, we established a gating strategy (**Figure 1**). Then, we analyzed whether these cell subsets differed in controlled and uncontrolled HAP cases. As shown **Figure 2**, Tfh cells were significantly increased in uncontrolled HAP (15.47% ± 5.49) compared to that in controlled HAP (11.58% ± 4.76, P=0.0072) and healthy group (10.24% ± 3.50, *P*=0.002). No differences in Tfh cell populations were found between the controlled and healthy groups (*P*=0.6541). Then we chose the ratio of Tfh/Th as main endpoint. Notably, the ratio of Tfh/Th performed a significant increase in uncontrolled HAP (0.19 ± 0.08) compared to disease-controlled HAP (0.13 ± 0.06, *P*=0.0088) and healthy group (0.12 ± 0.05, *P*=0.0026). Again, the differences between the controlled and healthy groups were not statistically significant (*P*=0.6670). These data indicated that the ratio of Tfh/Th is increased in progressive HAP patients and may be helpful for rapidly recognizing severity in HAP.

### 3.3 The ratio of Tfh/Th is elevated in K.p-infected HAP patients

**Figure 3:**
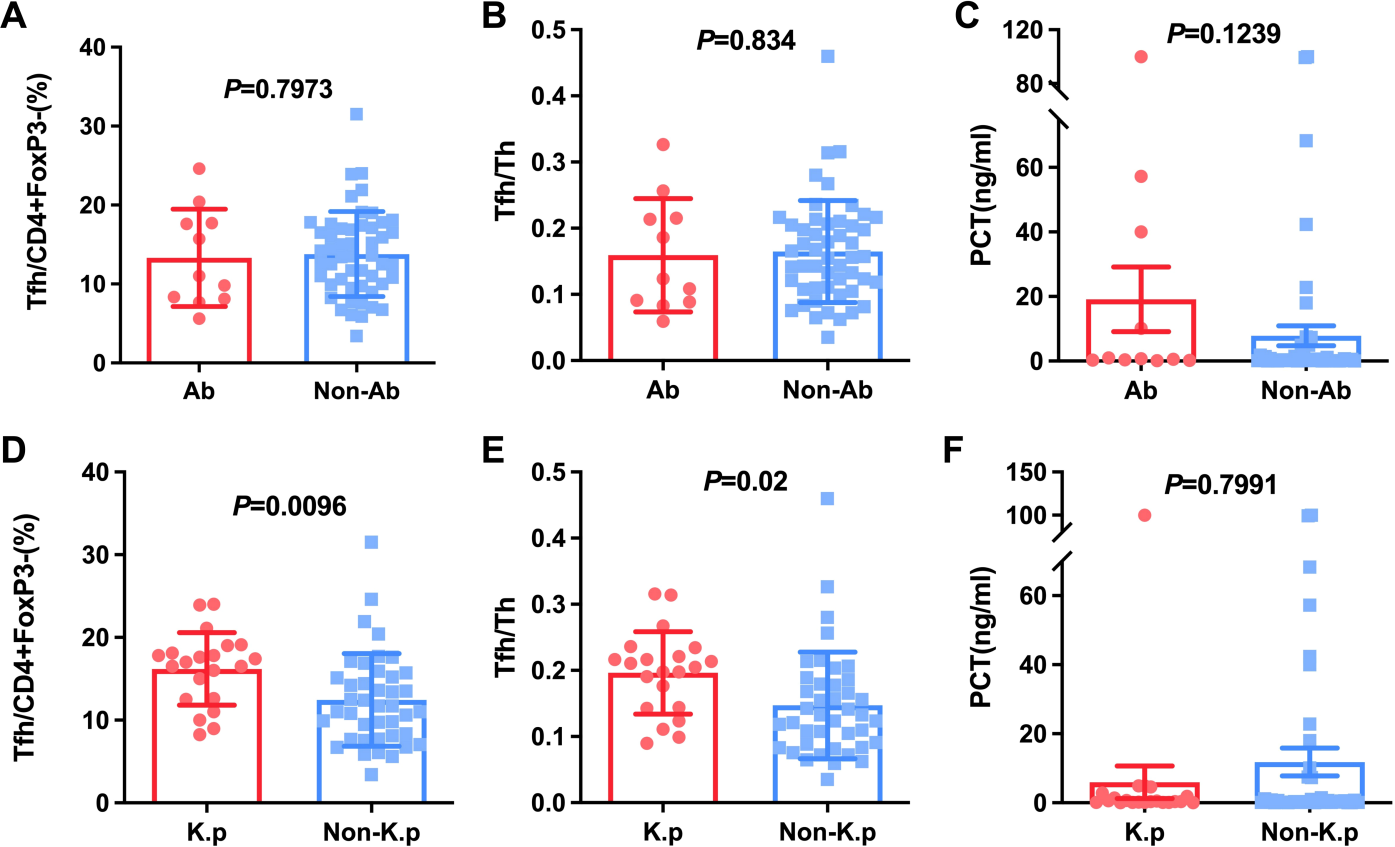
Comparison of Tfh and Tfh/Th and PCT in HAP patients with A.b and non-A.b infection **(A-C)**, with K.p and non-K.p infection **(D-F)**.

Next, we calculated Tfh cells to decide if these cells were relatively increased or decreased in special pathogenic bacteria-infected patients. The pathogenic bacteria were detected in 62 patients with HAP, of which including Klebsiella pneumoniae (K.p)-infected, (n=21), *Acinetobacter baumannii* (n=11), *Escherichia coli* (n=4), *Proteus mirabilis* (n=2), *Pseudomonas maltophilia* (n=6), *Pseudomonas aeruginosa* (n=8), *Pseudomonas cepacia* (n=2), *Staphylococcus aureus* (n=6), and *Aspergillus* (n=2). It was found that Tfh cells and the ratio of Tfh/Th were no significant difference in A.b-infected (13.33% ± 6.17, 0.16 ± 0.09) compared to non-A.b-infected patients (13.80% ± 5.39, 0.16 ± 0.08) with *P*=0.7973 and *P*=0.8340, respectively (**Figures 3A, B**). Interestingly, Tfh cells and the ratio of Tfh/Th were found to be significantly elevated in K.p-infected (16.20% ± 4.37, 0.20 ± 0.06) compared to non-K.p-infected patients (12.44% ± 5.61, 0.15 ± 0.08) with *P*=0.01 and *P*=0.02, respectively (**Figures 3D, E**). These data suggest that the immune response varied by different bacterial infections related to the Tfh.

### 3.4 Bloodstream infection and septic shock induced the rise of the ratio of Tfh/Th in subject with HAP

**Figure 4:**
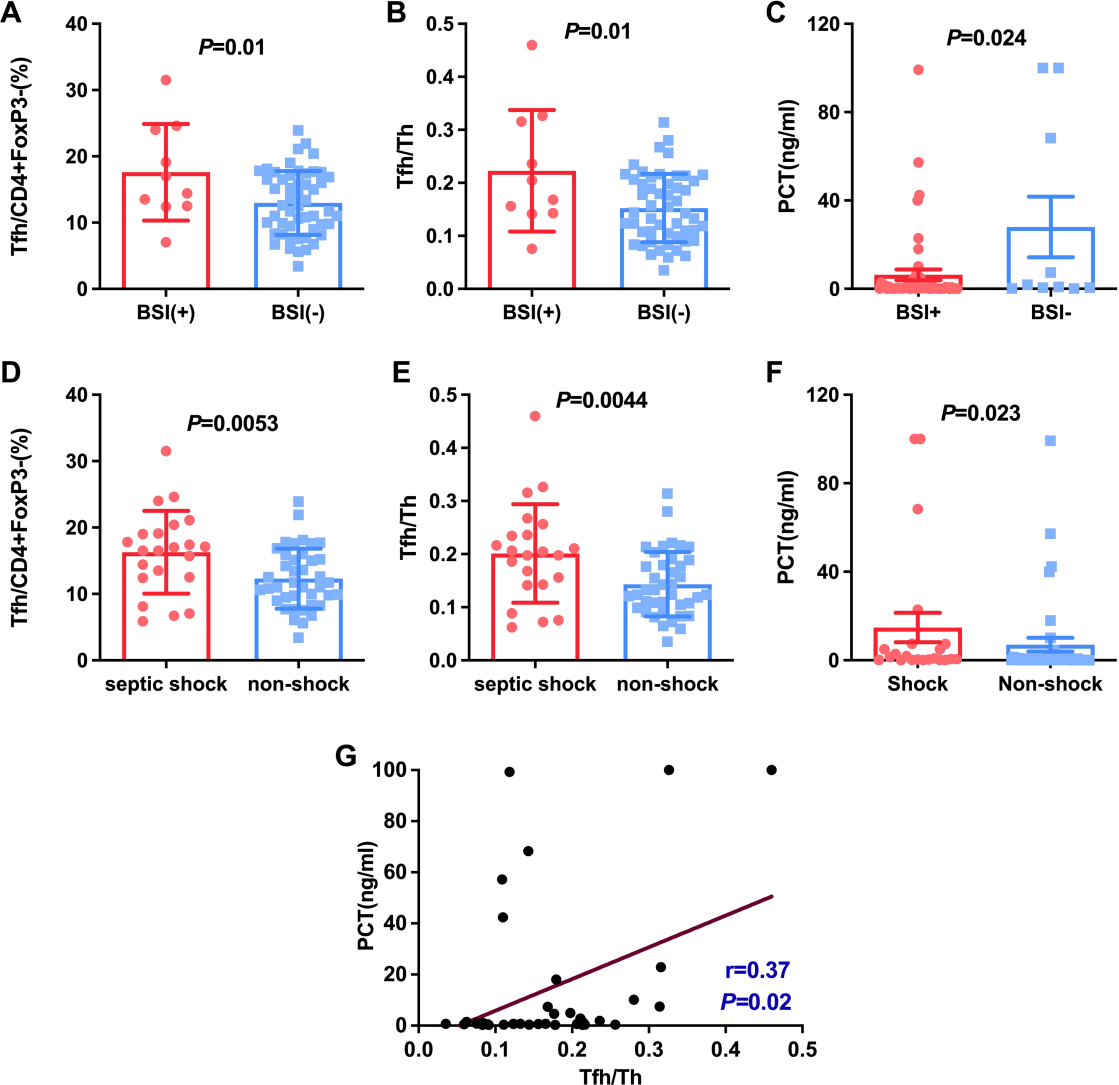
Comparison of Tfh and Tfh/Th and PCT in HAP patients with BSI and without BSI **(A-C)**, with septic shock and non-shock **(D-F)**. The correlation between PCT and the ratio of Tfh/Th in HAP was shown in **(G)**.

BSI is one of the most frequent lethal conditions managed in the ICU. We then investigated these different CD4+ cell subsets in HAP with BSIs and those without BSIs. As shown in **Figure 4A**, Tfh cells were significantly increased in HAP with BSIs compared with those without BSIs (16.20% ± 4.37 vs 12.98%± 4.82, *P*=0.01). Also, the ratio of Tfh/Th showed a significantly increase in patients with BSIs than in those without BSIs (0.22 ± 0.11 vs 0.15 ± 0.06, *P*=0.01) (**Figure 4B**). Moreover, Septic shock (SS) is one of the most common causes of death in HAP. Actually, there were some patients with suspected bloodstream infection in spite of negative blood culture, and they also display typical septic shock. We then investigated these different CD4+ cell subsets in HAP with SS and those without SS. As shown in **Figure 4D**, Tfh cells were significantly increased in HAP with Septic shock compared with those without Septic shock (16.29% ± 6.22 vs 12.30%± 4.52, *P*=0.005). Also, the ratio of Tfh/Th showed a significantly increase in patients with Septic shock than in those without Septic shock (0.20 ± 0.09 vs 0.14 ± 0.06, *P*=0.004) (**Figure 4E**). These data suggest that hemodynamic instability and systemic inflammation induced by severe sepsis amplify Tfh/Th imbalance, and the immune response caused by bacteria entering the blood would further mediate a more serious Tfh/Th imbalance.

Considering PCT usually was regarded as a marker for individuals with a bacterial infection (9, 10). We studied the relationship between PCT and Klebsiella, bloodstream infections as well as septic shock. The results indicated that PCT level in patients with BSIs and septic shock was significantly higher than patients without septic shock and BSIs (*P* < 0.05, **Figures 4C, F**). However, there were no significant differences in PCT level between K.p-infected and non-K.p-infected patients (**Figure 3F**), and the same results were observed in the A.b and non-A.b groups (**Figure 3C**). We next determined whether the ratio of Tfh/Th was correlated to PCT in subjects of HAP with PCT>0.25 μg/L. As shown in **Figure 4G**, Spearman’ s correlation test showed a good correlation between the ratio of Tfh/Th with PCT level (r=0.370, *P*= 0.02).

### 3.5 The combination of Tfh/Th and PCT shows better value in predicting deteriorated HAP than PCT alone

**Figure 5:**
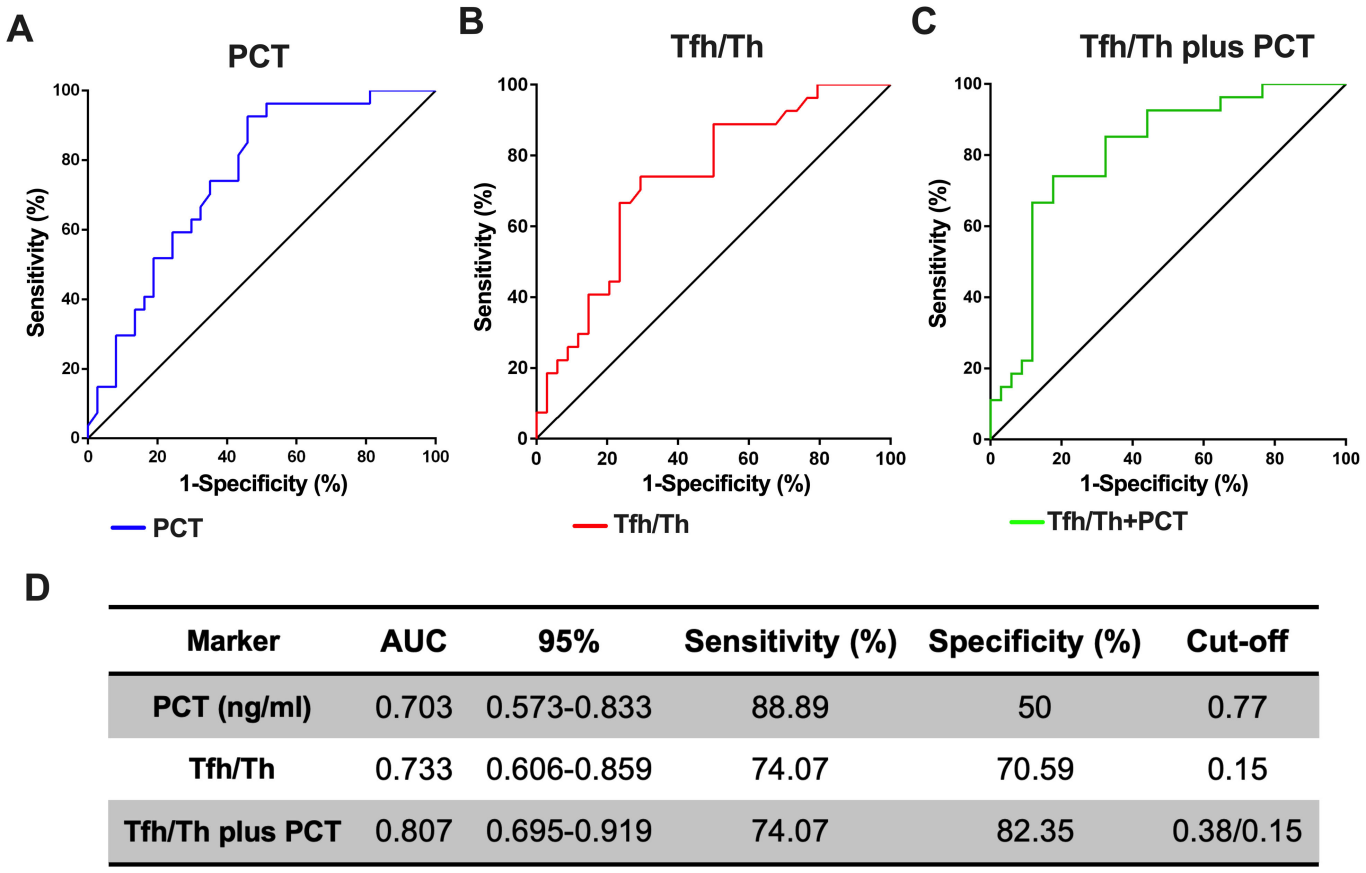
The combination of Tfh/Th and PCT is a better predicting biomarker for the deterioration of HAP. The ROC curves for PCT **(A)** and Tfh/Th **(B)** and Tfh/Th plus PCT **(C)** were shown. The 95% CI, sensitivity, specificity and cut-off for Tfh/Th, PCT and Tfh/Th plus PCT were shown in **(D)**.

We next hypothesized whether the ratio of Tfh/Th could complement to predict the progression of HAP. PCT alone had an Area under the curve (AUC) of 0.703 (95% CI, 0.57-0.83), with a sensitivity of 88.89% and a specificity of 50% respectively, when the critical value was 0.77 (**Figure 5A**). Tfh/Th alone had an AUC of 0.733 (95% CI, 0.61-0.86), with a sensitivity of 74.07% and a specificity of 70.59% respectively, when the critical value was 0.15 (**Figure 5B**). As expected, the Tfh/Th and PCT combination had an AUC of 0.807 (95% CI, 0.70-0.92), with a sensitivity of 74.07% and a specificity of 82.35% to predict the development of deteriorated HAP (**Figure 5C**). These data indicated that the addition of Tfh/Th could increase the potential of PCT as a biomarker to predict the deterioration of HAP (**Figure 5D**).

### 3.6 The ratio of Tfh/Th influenced the survival of HAP patients

**Figure 6:**
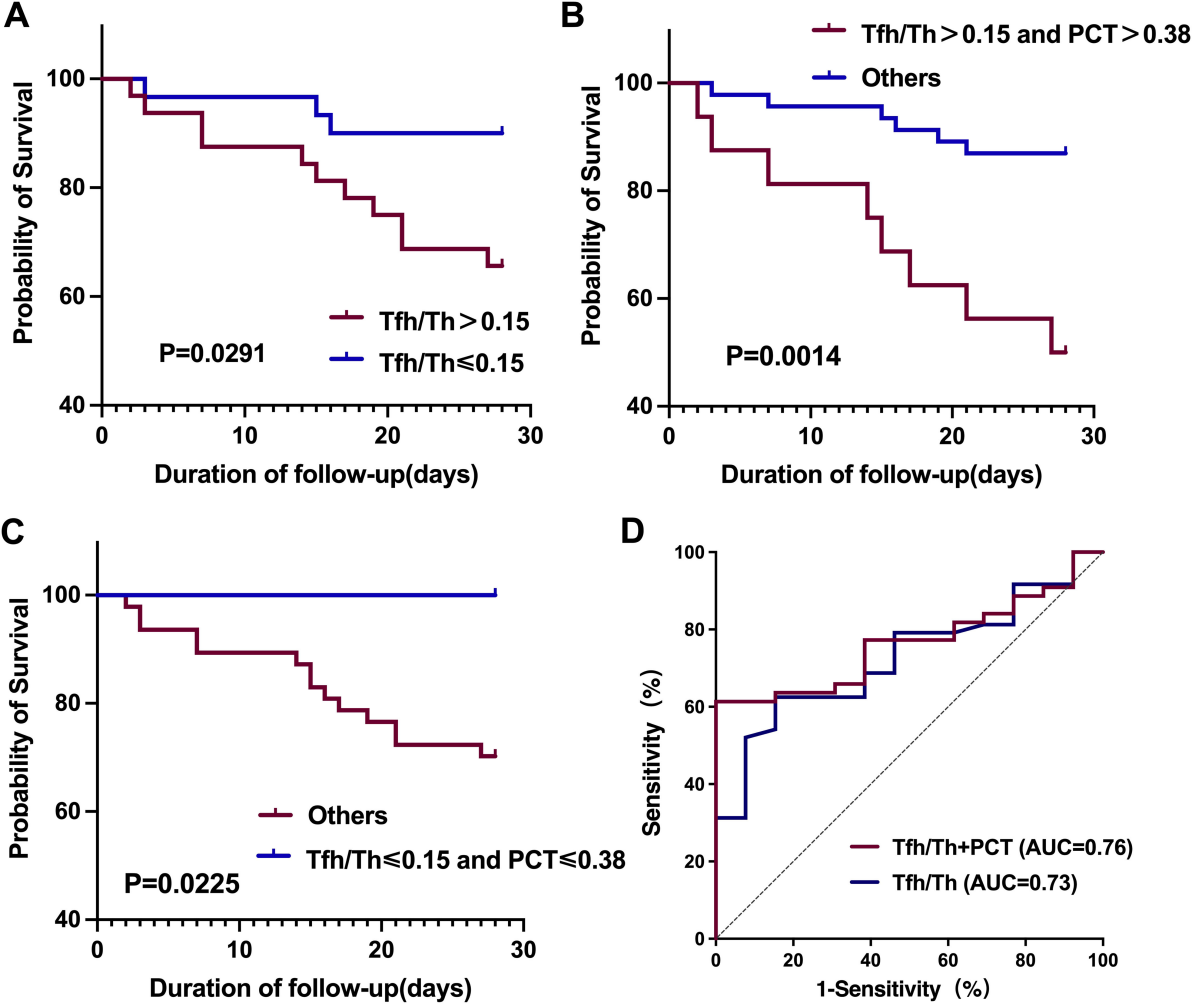
Kaplan-Meier survival curves and ROC curves for the 28-day mortality of HAP patients based on Tfh/Th ratios and PCT levels. **(A)** Kaplan-Meier survival curves comparing patients with high versus low Tfh/Th ratios, using a cutoff value of 0.15. **(B, C)** Kaplan-Meier survival curves assessing the combined prognostic impact of Tfh/Th ratios and PCT levels, with a PCT cutoff of 0.38 ng/m. Statistical significance was determined using the log-rank test. **(D)** ROC curves of Tfh/Th and Tfh/Th combined with PCT for predicting 28-day mortality.

Lastly, we investigated the prognostic value of Tfh/Th in HAP. Given the critical value of Tfh/Th in predicting deteriorated HAP, we divided the individuals into two groups by the median value of 0.15. By Kaplan-Meier survival analysis, the 28-day mortality of HAP patients with high (>0.15) and low (≤0.15) Tfh/Th ratios was assessed after the test day. As shown in **Figure 6A**, HAP patients with a high Tfh/Th ratio (>0.15) had a decreased survival rate in 28 days, compared to patients with a low Tfh/Th ratio (≤ 0.15). These data suggest that the ratio of Tfh/Th could be used as a predictor of survival in HAP.

In addition, we investigated the prognostic value of the combination of Tfh/Th ratios and PCT levels. We found that the HAP patients with high Tfh/Th ratio (>0.15) along with high PCT level (>0.38 ng/mL) had worse prognosis compared with the others (**Figure 6B**). Conversely, the HAP patients with low Tfh/Th ratio (≤0.15) along with low PCT level (≤0.38 ng/mL) had better survival than the others (**Figure 6C**). Furthermore, as shown in **Figure 6D**, we used ROC curve analysis to evaluate the predictive value for 28-day mortality and found that the area under the curve (AUC) for Tfh/Th combined with PCT (0.764, 95% CI, 0.644-0.884) was slightly larger than that for Tfh/Th alone (0.726, 95% CI, 0.592-0.860).

## 4 Discussion

HAP is a significant health burden worldwide. Immune dysregulation is reported to be closely associated with HAP in ICU patients (11). Patients in ICU usually have received various invasive treatments which could lead to an excessive inflammatory response with altered cell-mediated immunity. The dysregulated immune response can subsequently increase susceptibility to infection and cause HAP (11). In particular, the immune response regulation in HAP depends on complex interactions between alveolar macrophages, polymorphonuclear leukocytes, immune cells and local production of both pro- and anti-inflammatory cytokines as well as vascular adhesion molecules (12). Tfh cells play an indispensable role in forming and maintaining germinal centers for ongoing immune responses. Current research on the relationship of HAP with CXCR5+CD4+T cells is relatively scarce. Our current study has identified a Tfh cell subset (CD4+FoxP3-CXCR5+ cells) that is increased in progressive pneumonia and K.p-positive pneumonia. Further analysis has also shown that Tfh/Th can be a better diagnostic biomarker when assessing the severity of HAP than PCT. The findings of our study reveal the involvement of CD4+ Tfh cells in HAP and further provide a functional predictive value for the prognosis of HAP.

K.p is the leading cause of severe respiratory tract infections, and the mortality rate of K.p-induced pneumonia can exceed 50% (13). In addition, the increasing multidrug-resistant K.p strains pose a major medical problem worldwide (14–16). In this study, we also found that the patients with HAP were mainly infected with K.p(n=21). K.p has two distinctive subgroups, the classic K.p and the hypervirulent K.p. The classic K.p is notoriously known to gain antimicrobial resistance, while the hypervirulent K.p has an even higher antibiotic-resistant rate and causes more severe diseases (17). Therefore, the identification of K.p-infection in pneumonia diagnosis is essential for the treatment of the disease. In the current study, we have identified that Tfh/Th can be used to differentiate K.p and non-K.p infected pneumonia. It would be valuable to investigate if this biomarker can be used alone or in combination with other diagnostic markers to facilitate the identification of K.p-infected pneumonia. In addition, CD4+FoxP3-CXCR5+ cells are a subgroup of Tfh cells essential for developing and maintaining B cell immune responses (18).Therefore, it would be warranted to study if Tfh cells are involved in anti-K.p immune response and the findings will provide valuable information for developing better treatment strategies against K.p infection.

In ICU, BSI is another common complication in addition to HAP. Risk factors associated with BSI are multi-faced, including the patient’’ s underlying conditions and therapeutic, microbial and environmental factors (19). For HAP patients with BSIs, the immune system is further altered, and the balance between protective immunity and harmful hyper-inflammation is hard to be achieved (20). Specifically, this immune imbalance is particularly pronounced in patients with nosocomial bloodstream infections caused by Klebsiella pneumoniae, and it significantly increases the risk of mortality (21). The close relation of HAP and BSI to the dysregulated immune system explains that the change in the Tfh/Th immune cell ratio could serve as a biomarker when HAP is complicated with BSI.

Septic shock (SS) is a severe complication often accompanying hospital-acquired pneumonia (HAP) and remains a leading cause of mortality in ICU patients (22). The immune dysregulation associated with septic shock significantly exacerbates the host’s inability to achieve a balanced immune response (23). Our study has demonstrated a marked increase in the Tfh/Th ratio in HAP patients with septic shock compared to those without, suggesting a substantial immune imbalance. This aligns with previous reports indicating that hemodynamic instability and systemic inflammation induced by severe sepsis amplify immune dysfunction (24). The elevation in Tfh cells in septic shock reflects the heightened activation of CD4+ T cells in response to bacterial invasion and subsequent inflammation. Tfh cells are known to support B cell differentiation and antibody production, which are critical for combating infections (25). However, their dysregulated increase, as evidenced by the elevated Tfh/Th ratio, may contribute to the exaggerated inflammatory response observed in septic shock (26). This imbalance could hinder effective pathogen clearance while promoting immune-mediated tissue damage. Given the complexity of immune responses in septic shock, identifying reliable biomarkers for immune dysregulation is imperative for improving patient outcomes. In our study, the combination of Tfh/Th ratio and PCT levels showed strong prognostic value in HAP, even when complicated by septic shock. These findings suggest that integrating Tfh/Th with other clinical markers could enhance the early detection and management of immune dysregulation in septic shock. Future research is warranted to further explore the mechanistic role of Tfh cells in the immune pathogenesis of septic shock and their potential as therapeutic targets.

There is currently not enough research on bacterial pneumonia-related markers that can represent a definite diagnosis of bacterial pneumonia (27). In addition, the discovery of biomarkers that can differentiate between viral and bacterial pneumonia is also of great importance to avoid the unnecessary use of antibiotics (28). In an inpatient setting, CRP, WBC and PCT are usually part of the diagnostic workup (9, 29, 30). However, the changes in these parameters are not always specific to predicting causative pathogen. Several new biomarkers like MxA1, HMGB1 and CXCR5+CD8+ T cells have shown more promising results at preclinical and clinical levels. However, the rise or drop of a single marker is still not accurate enough to predict viral/bacterial pneumonia (6, 31). In the current study, we have identified another CD4 co-receptor expressing the Tfh cell subclass as a promising biomarker for assessing the deterioration of HAP. Based on the ROC analysis, the Tfh/Th and PCT combination is a better marker than the classical PCT alone. A combination of two or more markers gives better predictive accuracy. In addition, our study has also shown that the critical value of Tfh/Th can be an excellent prognostic marker for HAP survival.

Our current and previous study have shown that CD4+ Tfh cells are associated with HAP. However, the underlying mechanisms remain to be further investigated. The involvement of Tfh cells in the pathogenesis of bacterial pneumonia is general under investigation. Tfh cells are typically derived from CD4+ T cells and play a significant role in inflammation and immune regulation (32, 33). These cells have several cell surface markers, including CXCR5, PD1, BCL6 and ICOS, and they can be found in the B cell zone of secondary lymphoid organs and in circulation (34–36). Tfh cells are essential for developing and maintaining B cell immune responses by promoting B cell proliferation and maturation with co-stimulating signals (4). The relationship between Tfh cells and germline center B cells is positively related, and the failure of germline center formation and defects of antibody production observed in the absence of Tfh cells (37). It is also noted that Tfh cells play a key role in regulating the function of CD8+ T cells through secreting IL-21, such as enhancing the cytotoxicity and surface molecule expression of CD8+ T cells (36). Given the importance of Tfh cells in initiating and maintaining cellular immune responses, it is rational to speculate that the elevation of CD4+ Tfh cells in bacterial pneumonia is accompanied by host immune responses after pathogen infection in the low respiratory sites. Although beyond the scope of our current study, it is interesting to investigate the importance of Tfh cell function in bacterial pneumonia.

## 5 Conclusions

Tfh/Th ratio is useful for identifying the exacerbation of the patients with HAP and increased Tfh/Th ratio indicates progressive HAP, especially K.p infection. The combination of these cells and PCT could be used as a better predictor of impaired outcome than PCT alone. The findings in our current study provide not only better understanding of the involvement of CD4+ Tfh cells in HAP, but also identify a potential prognostic biomarker for HAP.

## Data Availability

The raw data supporting the conclusions of this article will be made available by the authors, without undue reservation.
